# Novel Hemizygous Mutations of *TEX11* Cause Meiotic Arrest and Non-obstructive Azoospermia in Chinese Han Population

**DOI:** 10.3389/fgene.2021.741355

**Published:** 2021-09-21

**Authors:** Zhiyong Ji, Chencheng Yao, Chao Yang, Chuan Huang, Liangyu Zhao, Xia Han, Zijue Zhu, Erlei Zhi, Nachuan Liu, Zhi Zhou, Zheng Li

**Affiliations:** ^1^State Key Laboratory of Reproductive Medicine, Nanjing Medical University, Nanjing, China; ^2^Department of Andrology, The Center for Men’s Health, Urologic Medical Center, Shanghai General Hospital, Shanghai Jiao Tong University School of Medicine, Shanghai, China; ^3^The Human Sperm Bank, Reproductive and Genetic Hospital of CITIC-Xiangya, Changsha, China; ^4^School of Life Sciences and Technology, Shanghai Tech University, Shanghai, China

**Keywords:** *TEX11*, novel mutation, male infertility, meiotic arrest, non-obstructive azoospermia

## Abstract

Testis-expressed gene 11 (*TEX11*) mutation has been associated with non-obstructive azoospermia (NOA) and meiotic arrest. An analogous mutation of *TEX11* in the mouse impairs meiosis and can be rescued by *in vitro* expansion of SSCs and gene therapy. However, a lack of genetic screening of a large cohort of Asian patients (including pedigree analysis) and proper functional evaluation limit the clinical application of *TEX11* mutation screening. Thus, we performed whole-exome sequencing (WES) in 479 patients with NOA and identified three novel mutations (two splicing mutations and one missense mutation) in *TEX11* in three pairs of siblings from three families and four novel pathogenic mutations (three frameshift mutations and a non-sense mutation) of *TEX11* in four sporadic NOA-affected cases. Novel variants among family members were segregated by disease phenotype, and all the seven mutations were predicted to be pathogenic. Histological analysis showed that three patients with *TEX11* mutations underwent meiotic arrest. The four mutations that resulted in protein truncations and defective meiosis-specific sporulation domain SPO22 were validated by Western blot. In total, we find seven of 479 patients of NOA (1.5%) carrying *TEX11* mutations. Our study expands the knowledge of mutations of *TEX11* gene in Asian patients with NOA. The high prevalence and X-linked inherited mode indicated that *TEX11* might be included in genetic screening panels for the clinical evaluation of patients with NOA.

## Introduction

The etiology of male infertility is complex. There are congenital causes like Y chromosome microdeletions, acquired causes like varicocele. And 30–50% of cases are idiopathic (Agarwal et al., 2021). The same cause of male infertility can result in heterogeneous phenotypes, which increases the difficulties for the diagnosis and treatment. The most severe phenotype is azoospermia (complete lack of spermatozoa in the ejaculate), affecting 1% of men (Stephen and Chandra, 2006). Azoospermia is typically classified into two subgroups: obstructive azoospermia (OA) with normal sperm production, fertility of which can be achieved through surgery, accounting for 30% of all patients; non-obstructive azoospermia (NOA), a spectrum of testicular disorders resulting in spermatogenic failure, and complete lack of sperm in the ejaculate, accounting for 70% of all patients (Agarwal et al., 2021).

Compared with OA, success rates of testicular sperm retrieval in men with NOA are substantially lower, leaving nearly half of patients with the option of artificial insemination with donor sperm or adoption (Corona et al., 2019). With this regard, the deep understanding of molecular mechanisms for NOA is the key to develop new therapies. Genetic defect is most likely the underlying cause of the severe forms of spermatogenic impairment such as severe oligozoospermia or azoospermia (Krausz and Riera-Escamilla, 2018). Current genetic standards for the diagnosis of severe male infertility include testing for numerical and structural chromosomal aberrations and deletions of the AZF regions on the Y chromosome. These approaches, however, yield a low diagnosis rate (10–20%) (Tüttelmann et al., 2018). The high sensitivity, high resolution, and low cost make the next-generation sequencing (NGS) widely applied in the genetic association studies. By using NGS technologies to perform more unbiased genomic studies, we identified infertility-associated mutations of numerous genes (e.g., *SYCP2*, *SYCP3*, and *TEX11*) (Miyamoto et al., 2003; Yatsenko et al., 2015; Schilit et al., 2020).

Testis-enriched genes related to spermatogenesis are commonly located on sex chromosomes in males (Yang et al., 2008; Krausz and Riera-Escamilla, 2018). *De novo* or rare mutations in the sex chromosomes of the males are more inclined to cause clinical phenotypes due to lack of compensatory allele. *TEX11* is one of the X-linked genes and considered to be indispensable for normal spermatogenesis, especially meiosis playing a role in maintaining synaptonemal complex and promoting cross over. TEX11 is enriched in late spermatocytes and spermatids in mice (Wang et al., 2001), disruption of which in knock-out mice causes meiotic arrest and azoospermia (Yang et al., 2008). Yatsenko et al. (2015) found five *TEX11* pathogenic mutations in seven of 289 men with azoospermia (2.4%) and in 33 patients (15%) diagnosed with meiotic arrest. The phenotype of these patients is similar to that of *Tex11*-deficient male mice. By clustered regularly interspaced short palindromic repeats–CRISPR-associated endonuclease 9 (CRISPR-Cas9)-mediated homology-directed repair (HDR), a patient-derived *TEX11* monogenic mutation that causes human azoospermia was verified and corrected in mice (Wang et al., 2021). However, these mutations are rarely reported in Asian populations, indicating that the mutations of *TEX11* may differ in various races.

In this study, we performed WES with blood samples from 479 patients with NOA. Seven novel mutations of *TEX11* were found (three familial and four sporadic cases) and validated by Sanger sequencing. The testis tissues of probands were evaluated by hematoxylin-eosin (HE) staining and immunostaining to further explore the role of TEX11.

## Materials and Methods

### Patients

Participants were recruited from the Department of Andrology, Urologic Medical Center, Shanghai General Hospital, Shanghai Jiao Tong University. Patients were diagnosed with NOA by means of semen analysis performed according to the guidelines of the World Health Organization. Patients with a history of non-genetic causes for spermatogenic impairment (e.g., trauma, surgery, or medication), obstructive azoospermia (e.g., CBAVD), and with chromosomal abnormalities or Y chromosome microdeletions were excluded from the study. The work was approved by the Institutional Review Board of Shanghai General Hospital (Permit Number: 2020SQ199). Written informed consents for testicular biopsies were obtained from each participant. The probands in families P5648, P6825, and P7583 underwent the microsurgical testicular sperm extraction (mTESE) procedure at our center, and no sperm was found. Other patients that have undergone testicular biopsy in other hospital without finding of sperm would not prefer a reoperation.

### Whole Exome Sequencing and Data Analysis

Genomic DNA was extracted from peripheral blood mononuclear cells of patients with NOA using the TIANamp Blood DNA Kit (TIANGEN Biotech, Beijing, China) according to the instructions of the manufacturer. DNA was fragmented through Covaris focused ultrasonication. Known exons and exon–intron boundary sequences were captured using xGen Exome Research Panel (Integrated DNA Technologies, Coralville, IA, United States), and sequencing DNA libraries were prepared following the instructions of the manufacturer. Sequencing was performed on a HiSeq X10 platform (Illumina, San Diego, CA, United States). Sequencing reads were aligned to the human genome (GRCh37/hg19) using Burrows–Wheeler Aligner (BWA). Both SNVs and indels within the captured coding exonic intervals were called using GATK, Platypus, VarScan, LoFreq, FreeBayes, SNVer, SAMtools, and VarDict. The variants were filtered and annotated using the ANNOVAR software.

Genetic variants with allele frequencies higher than 1% according to the ExAC Browser and 1000 Genomes Project were excluded, and the intronic, upstream, and downstream variants were removed. Non-sense, frameshift, essential splice-site, and potentially deleterious missense (SIFT, PolyPhen-2, and Mutation Taster) variants were saved for further analysis. Genes with two alleles with potentially deleterious missense mutations or loss-of-function (LoF) mutations were retained because autosomal recessive and X-linked inheritance were assumed for MA. Also, we compared candidate genes with known pathogenic genes for azoospermia in mice^1^ and testis-enriched genes in the database^2^. The aforementioned sequencing and bioinformatic analyses were conducted by the Nuprobe Company (Shanghai, China). All positive calls were further investigated and confirmed *via* Sanger sequencing. The primers are shown in online Supplementary Table 1.

We used the Pfam database to search for protein domains in TEX11 (Mistry et al., 2021). The TPRpred method was used to identify the tetratricopeptide repeat (TPR) regions in the protein (Gabler et al., 2020).

### Histological Analysis

Tissue samples were fixed overnight in 4% paraformaldehyde followed by dehydration, embedding, and sectioning following standard protocols. Sections were subjected to H&E staining for routine analysis.

For immunofluorescence, sections were deparaffinized, rehydrated in a descending ethanol row, and rinsed with water. Heat-induced epitope retrieval and immunofluorescence were performed as described previously (Yao et al., 2020). The sections were blocked with 6% normal donkey serum for 1 h at room temperature, followed by incubation overnight with anti-SYCP3 (dilution: 1:25, catalog number: AF3750; R&D Systems, Minneapolis, MN, United States), anti-γH2AX (dilution: 1:400, catalog number: 2668445; Merck Millipore, Billerica, MA, United States), anti-DMC1 (dilution: 1:200, catalog number: sc-373862; Santa Cruz Biotechnology, CA, United States), and PNA antibodies (dilution: 1:400, catalog number: L21409; Life Technologies, Waltham, MA, United States) at 4°C in a humidity chamber. The sections were washed thrice with PBS-Tween, and incubated with highly cross-adsorbed secondary antibody conjugated with Alexa Fluor 488 or Alexa Fluor 555 (dilution: 1:400, Life Technologies) for 1 h at room temperature. After three washes, the nuclei were stained with Hoechst 33342. The images were captured by fluorescence microscope (Leica, Wetzler, Germany).

### Plasmid Construction and Mutagenesis

Human *TEX11* cDNA (GenBank No. NM_001003811) was cloned in the pcDNA vector with MYC-tag by homologous recombination *via* the ClonExpress^TM^ one-step cloning kit (Vazyme, C112). The cDNA obtained from the testis of patients with OA were used as the template for PCR amplifications. The linearized vector was obtained by digesting the circular vector with double restriction enzyme (*Kpn*I-HF^®^ and *Eco*RI-HF^®^) digestion. All deletions and substitution mutants from the *TEX11* expression constructs were generated by PCR. The PCR was performed with the PrimeSTAR system (Takara, Beijing, China) using the following conditions: 98°C for 5 min, 18 cycles of 98°C for 10 s, 60°C for 5 s and 72°C for 270 s, 7 min at 72°C and forever at 4°C. All constructs were verified by sequencing before the experiments. Then 0.1 μl of DMT Enzyme (TRANS^®^GD111-01 20,000 U/ml) was added to 10 μl of the reaction, mixed well, and incubated at 37°C for 1 h. The final reaction was transformed into competent cells. Colony, miniprep, and sequence were picked to check for mutation and any PCR-introduced errors. All primers used for plasmid construction are summarized in Supplementary Table 2.

#### Cell Culture and Transfection

Human embryonic kidney 293 (HEK293) cells (National Collection of Authenticated Cell Cultures, Beijing, China) were cultured in Dulbecco’s modified Eagle’s medium (DMEM; Gibco, Beijing, China) supplemented with 10% fetal bovine serum (Gibco) and 1% penicillin–streptomycin at 37°C in 5% CO_2_ atmosphere. To express the mutant proteins, the plasmids mimicking *TEX11* mutations were transfected into HEK293 cells with Lipofectamine 3000 reagent (Invitrogen).

### Western Blotting

Forty-eight hours after transfection, the cells were lysed in RIPA lysis buffer (Beyotime, Shanghai, China) for 5 min on ice, followed by centrifugation at 13,000 × *g* for 5 min at 4°C to isolate the protein, which was quantified by bicinchoninic acid (BCA) and electrophoresed on 10% sodium dodecyl sulfate polyacrylamide gels and were transferred to a 0.45-μm nitrocellulose filter membrane (NC, GE Healthcare Life Sciences, PA, United States). The membranes were blocked with 5% skim milk in PBST buffer. After washing three times with PBST for 10 min, anti-c-Myc rabbit monoclonal antibodies (71D10, Cell Signaling Technology, Danvers, MA, United States) were added to detect TEX11-c-Myc proteins at 1:1,000 dilution using anti-rabbit IgG HRP (1:5,000; CST; #7074) and ECL (Millipore) in an AI600 imager (GE healthcare). The densitometry was analyzed using the AI600 software. The housekeeping protein, GAPDH, was used for Western blot normalization.

## Results

### Clinical Data

We investigated the genetic causes of male infertility in a cohort of 479 patients with NOA using WES, and pathogenic variants of *TEX11* were identified in 14 patients (four sporadic and 10 from three families). The patients were diagnosed with NOA during routine semen analyses and testicular biopsy (in our hospital or other hospital). The results of physical examinations showed that the infertile patients with pathogenic variants in *TEX11* were healthy. These patients did not have a history of cryptorchidism, hypogonadism, cancer, drinking, or smoking. Physical examination showed normal development of penis, epididymis, prostate, scrotum, and vas deferens without signs of varicocele. The volume of their testes and laboratory examination such as sex hormone levels were recorded, and most of the data were within the normal range. All patients have 46, XY karyotypes without microdeletions in the Y chromosome. The clinical data for the patients with pathogenic variants in *TEX11* are summarized in Table 1.

**TABLE 1:** Clinical characteristics of the Chinese study subjects at the clinical assessment.

**Parameters**	**Age (years)**	**Left testicular volume (ml)**	**Right testicular volume (ml)**	**FSH (IU/L)**	**LH (IU/L)**	**Testosterone (μg/L)**	**Semen volume (ml)**	**Total sperm count**	**Karyotype**	**Y chromosome microdeletions**
P5648	26	10	12	6.4	5.7	7.6	1.8	0	N	N
P5648-B	23	10	10	7.8	4.8	6.2	2.1	0	N	N
P6825-II-1	41	12	12	3.8	4.1	3.5	1.6	0	N	N
P6825-II-7	36	12	15	10.2	6.2	4.6	1.7	0	N	N
P6825-II-9	34	12	12	8.6	7.4	5.5	2.3	0	N	N
P6825-II-13	33	10	12	7.5	5.5	5.1	1.6	0	N	N
P6825-III-17	18	10	12	10.1	7.5	5.7	1.9	0	N	N
P6825-III-18	17	12	12	4.7	6.8	6.3	1.6	0	N	N
P7583	33	15	15	10.7	7.9	3.9	3.2	0	N	N
P7583-B	29	12	15	7.9	6.4	4.7	2.5	0	N	N
P5048	26	12	15	4.8	3.8	5.1	1.8	0	N	N
P8122	30	12	12	8.4	5.8	4.6	1.7	0	N	N
P8251	27	10	12	5.5	4.2	3.7	2.1	0	N	N
P9225	31	12	12	9.8	6.5	6.1	2.4	0	N	N

*AZF, azoospermia factor; FSH, follicle-stimulating hormone; LH, luteinizing hormone; N, normal phenotype.*

**FIGURE 1:**
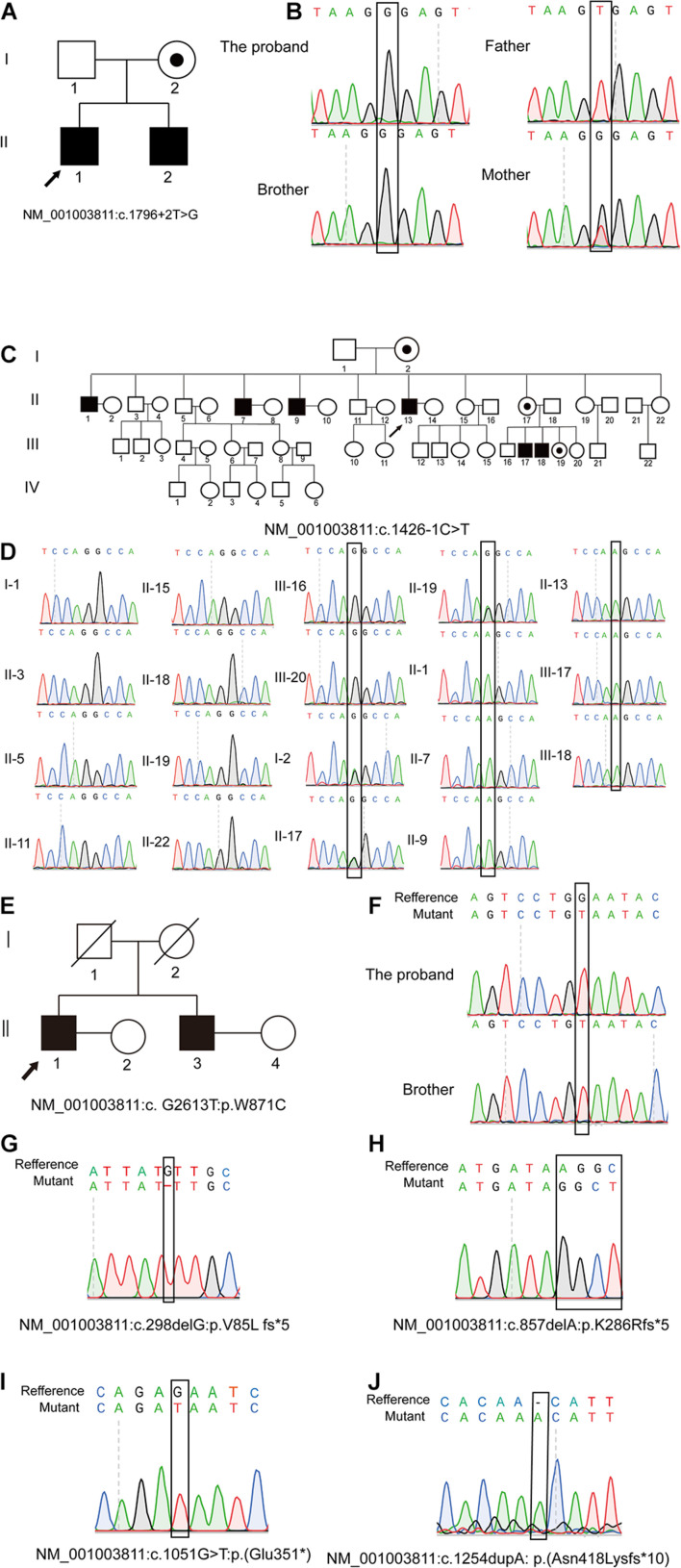
Pedigree of families and Sanger sequencing validation. Pedigrees of three families with X-linked congenital azoospermia. The arrowheads indicate the probands, Squares indicate males, circles indicate females, slashed symbols indicate deceased, black symbols indicate affected individuals, unfilled symbols indicate unaffected individuals, and unaffected, obligate carriers are represented by a dotted circle. **(A,B)** Pedigree of family P5648: The two affected brothers underwent whole-exome sequencing (WES) and their mother is obligate unaffected carrier. **(C,D)** Pedigree of family P6825: The proband II-1 underwent WES, while other family members underwent Sanger sequencing. Four brothers and two nephews (II-1; II-7; II-9; II-13; III-17; III-18; III-1; III-2) are affected individuals. The sister of the proband, niece, and mother are obligate unaffected carriers (I-2; II-17; III-19). Other family members do not carry the mutation (I-1, II-3, II-5, II-11, II-15, II-18, II-19; II-22; III-16; III-20). **(E,F)** Pedigree of family P7583: the proband and his affected twin brother underwent WES while their parents are deceased. **(G–J)** Four affected individuals underwent WES and validated by Sanger.

Specifically, P5648 and P5648-B are two affected brothers with healthy parents while their mother is a heterozygous pathogenic variant carrier; II-1, II-7, II-9, and II-13 of P6825 family are four affected brothers, while the two nephews III-17 and III-18 are also affected; P7583 and P7583-B are affected twin brothers with their parents passing away several years ago. The Sanger sequencing analysis of patients and their family members was performed. The hemizygous pathogenic variant NM_001003811: c.1796 + 2T > G in *TEX11* was confirmed in the sibling patients P5648/II:1 and P5648/II:2. Their mother is heterozygous pathogenic variant carrier (Figures 1A,B). NM_001003811: c.1426-1C > T were confirmed in patient P6825/II:1, P6825/II:7, P6825/II:9, P6825/II-13, P6825/III-1, P6825/III-17, P6825/III-18. Their mother, sister, and niece (I-2, II-17, III-19) are carriers of NM_001003811: c.1426-1C > T as a heterozygous variant. The blood samples that could be obtained from other family members’ (I-1, II-3, II-5, II-11, II-15, II-18, II-19, II-22, III-16, and III-20) were tested and found to be normal (Figures 1C,D). Family P7583 had two affected twin brothers carrying the same pathogenic variant NM_001003811: c.2613G > T: p.W871C (Figures 1E,F), whose parents passed away. The pathogenic variants were segregated in a phenotypic and familial manner, which is consistent with Mendelian inheritance.

The other four pathogenic variants NM_001003811: c.1051G > T: p.E351^∗^; NM_001003811: c.1254dupA: p.N418K fs^∗^10; NM_001003811: c.298delG: p.V85L fs^∗^5; NM_001003811: c.857delA: p.K286R fs^∗^5 were confirmed in patient P5048, P8122, P8251, and P9225 by Sanger sequencing, respectively (Figures 1G–J).

### *In silico* Analysis of the Novel Pathogenic Variants of Testis-Expressed Gene 11

**FIGURE 2:**
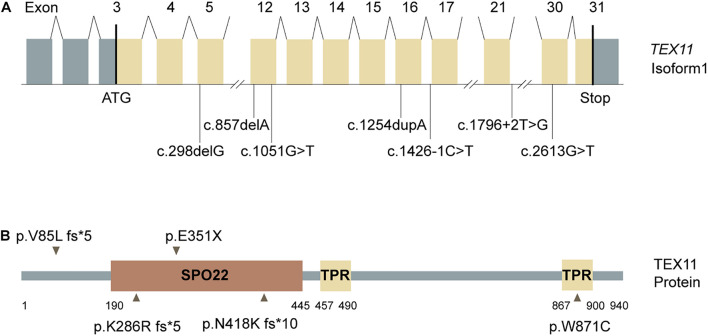
Testis-expressed gene 11 (*TEX11)* mutations detected in men with azoospermia. **(A)** The genomic structure of TEX11, with mutations mapped to isoform 1 (GenBank accession number, NM 001003811.1). Gray rectangles represent non-coding exons, and yellow rectangles represent coding exons. The coding sequence of the gene begins at nucleotides that encode a start codon in exon 3 and ends in exon 31 at a stop codon. All mutations detected in men with azoospermia are shown. **(B)** The predicted TEX11 domains with multiple tetratricopeptide repeat (TPR)–containing regions (amino acid positions 457–490 and 867–900), and a sporulation domain (SPO22) meiosis-specific motif (amino acid positions 190–445). Mutations in the coding region (black arrowheads) and splicing changes (black lines) are located in predicted domains.

We evaluated the novel pathogenic variants of *TEX11* through *in silico* analysis. First, we analyzed the frequency of novel *TEX11* variants. Frequency data, which were compiled from common databases (gnomAD, ExAC), indicated that the novel variants are not found in the population (Table 2), which is consistent with the prevalence of NOA. Next, we examined the evolutionary conservation of amino acids in sequences, which are possibly affected by the pathogenic variants. We aligned the amino acid sequences of *TEX11* from human to zebrafish and found that the amino acid encoded by the pathogenic variant is highly conserved across species (Supplementary Figure 1). In addition, we analyzed the pathogenicity of novel variants in *TEX11*. Most databases predicted that these novel variants were highly pathogenic (Table 2). There are five mutated sites located in the exons of *TEX11* (Figure 2A), leading to protein truncation or amino acid substitutions in the functional domains, and two mutations were predicted to result in splicing alterations (Figure 2B).

**TABLE 2:** *In silico* analysis of the novel pathogenic variants of *TEX11.*

**Patient no.**	**Change in coding DNA (NM_001003811)**	**Protein/RNA change**	**Phenotype**	**GnomAD minor allele frequency (%)**	**EXAC**	**Polyphen2**	**SIFT**	**Mutation taster**	**CADD score**
P5648	c.1796 + 2T > G	p.599K spl d	MA	NA	NA	NA	NA	Disease causing	NA
P6825	c.1426-1C > T	p.476A spl d	MA	NA	NA	NA	NA	Disease causing	5.2
P7583	c.2613G > T	p.W871C	NOA	NA	NA	NA	NA	Disease causing	NA
P5048	c.1051G > T	p.E351*	MA	NA	NA	Possibly damaging	Deleterious	Disease causing	NA
P8122	c.1254dupA	p.N418K fs*10	MA	NA	NA	NA	NA	Disease causing	NA
P8251	c.298delG	p.V85L fs*5	NOA	NA	NA	NA	NA	Disease causing	NA
P9225	c.857delA	p.K286R fs*5	NOA	NA	NA	NA	NA	Disease causing	NA

*MA, maturation arrest; NOA, non-obstructive azoospermia; NA, not available.*

### MA Phenotypes in Cases With Hemizygous Mutation in Testis-Expressed Gene 11

**FIGURE 3:**
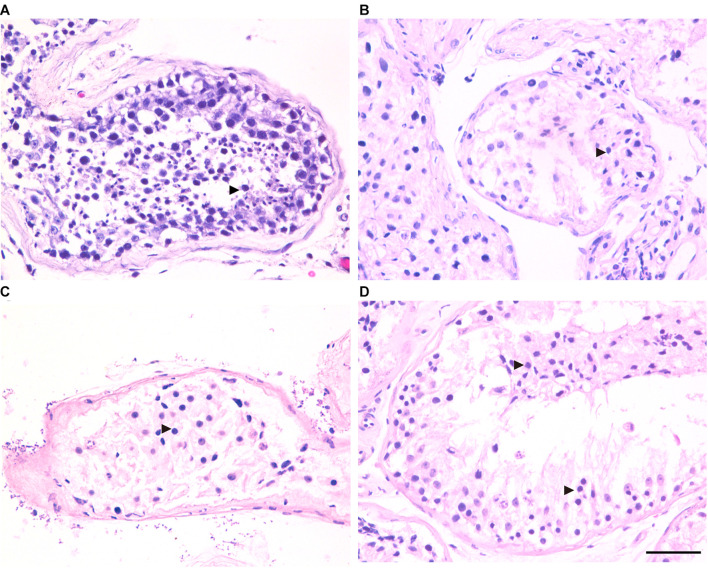
H&E staining of cross-sections of testis in affected patients and a patient with obstructive azoospermia (OA) as positive control. **(A)** Hematoxylin-eosin (H&E) staining of cross-sections of testicular biopsy from the patient with OA **(A)**, the P5648 **(B)**, the P6825 **(C)**, 7583 **(D)**. Scale bars = 50 μm in panel **(D)**. Black triangles indicate the spermatocytes in the testis.

**FIGURE 4:**
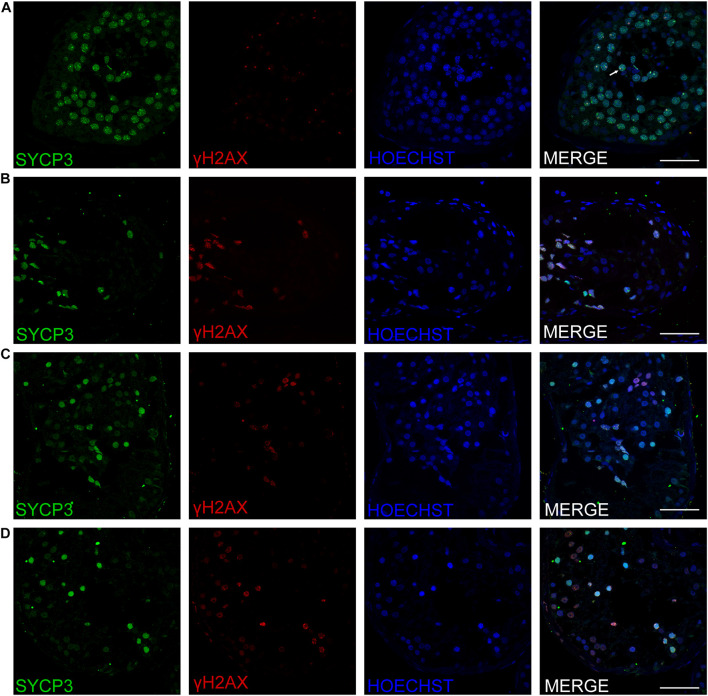
Expression of SYCP3 and γH2AX in the testis of affected patients and a patient with OA as positive control. Immunohistochemical staining showed the expression of SYCP3 (green) and γH2AX (red) in the testis of the patient with OA (positive control) **(A)**, the P5648 **(B)**, the P6825 **(C)**, 7583 **(D)**. Scale bars = 50 μm in panels **(A–D)**. The arrow indicates the XY body in the spermatocytes in the testis.

**FIGURE 5:**
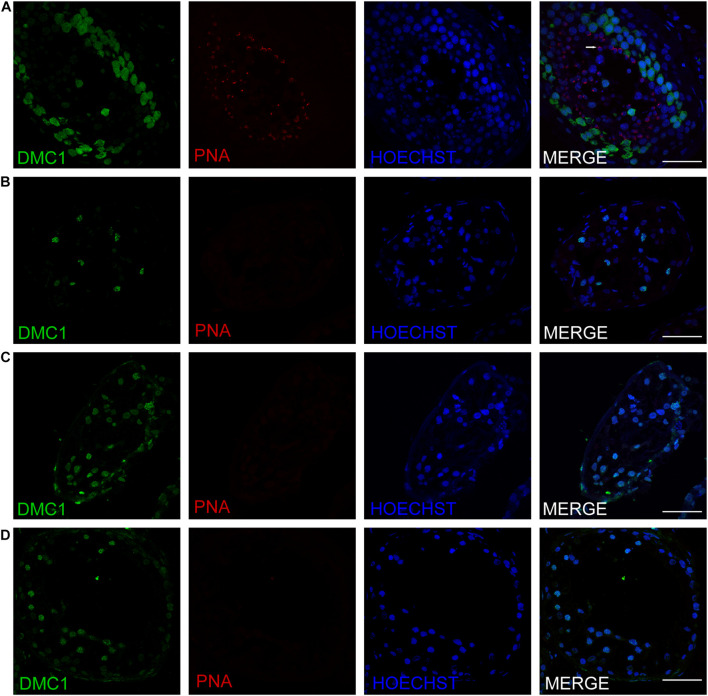
Expression of recombination proteins DMC1 and acrosomal marker PNA in the testis of affected patients and a patient with obstructive azoospermia as a positive control. Immunohistochemical staining showed the expression of DMC1 (green) and PNA (red) in the testis of the patient with OA as positive control **(A)**, the P5648 **(B)**, the P6825 **(C)**, 7583 **(D)**. Scale bars = 50 μm in panels **(A–D)**. Arrow indicates the acrosome of spermatids in the testis.

Three patients (P5648, P6825, and P7583) received testis biopsies in our hospital, and other patients had already undergone the same surgery in other hospitals, and no sperm was found (data not available). H&E analysis of testis of OA patient showed spermatids and different stages of male germ cells. Compared with normal controls, the testicular histology from patients showed a thicker basement membrane of seminiferous tubules and poorly developed spermatocytes. No post-meiotic round spermatids or mature spermatozoa were observed in the seminiferous tubules (Figure 3). The testis biopsies of the patients showed positive expression of DMC1 (indicating double-strand break repair), SYCP3 (components of the axial/lateral element), and γH2AX (a DNA double-stranded break marker), but negative for PNA (a marker of spermatids and spermatozoa) or XY body (maker of pachytene spermatocytes indicated by γH2AX staining), suggesting MA at spermatocytes (Figures 4, 5). Based on the histological analysis, the phenotypes caused by *TEX11* mutations were concluded to be meiotic arrest.

### Testis-Expressed Gene 11 Protein Expression in Different Kinds of Mutations

**FIGURE 6:**
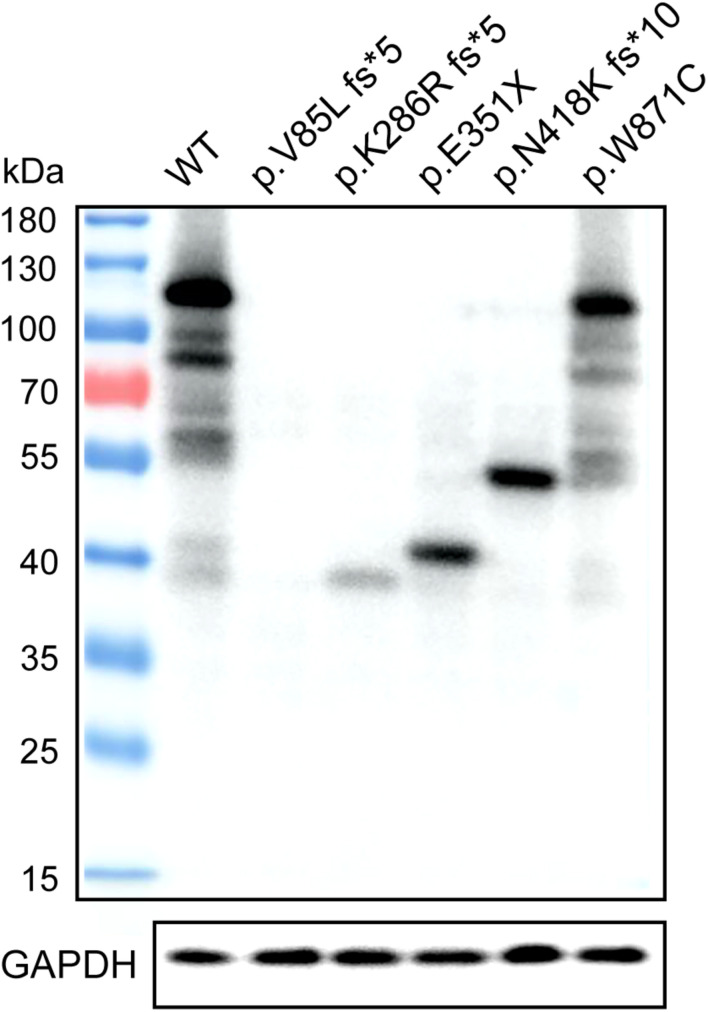
Expression of wild-type TEX11 and the mutant variant in HEK293 cells. Western blotting results stained for anti-Myc antibody with HEK293 cells transiently transfected with wild-type TEX11-Myc, TEX11-mutant (p.V85L fs*5)-Myc, TEX11-mutant (p.K286R fs*5)-Myc, TEX11-mutant (p.E351X)-Myc, TEX11-mutant (p.N418K fs*10)-Myc, TEX11-mutant (p.W871C)-Myc vectors. GAPDH served as a loading control, and molecular weight markers are shown on the left.

Western blotting was used to assess TEX11 protein expression in HEK293 cells that were transiently transfected with expression vectors, including a fusion construct of the wild-type TEX11 coding sequence (CDS) with a terminal Myc tag, and fusion constructs of the CDS for the p.V85L fs^∗^5 (P8251), p.K286R fs^∗^5 (P9225), p.E351X (P5048), p.N418K fs^∗^10 (P8122), p.W871C (P7583) mutant allele with a Myc tag as well as normal Myc-TEX11. Mutant protein of p.K286R fs^∗^5 (P9225), p.E351^∗^ (P5048), p.N418K fs^∗^10 (P8122), p.W871C (P7583) of TEX11 were detected as expected (approximately 110, 40, 47, 57, and 110 kDa, respectively) from cells with the expression of corresponding plasmids, while the expression of p.V85L fs^∗^5 (P8251, approximately 20 kDa) mutant protein was not detected (Figure 6).

## Discussion

The process of meiosis is highly conserved across eukaryotes, which is essential for the maintenance of biologic diversity and species survival. The abnormality of proteins involved in proper chromosome segregation and DNA recombination are generally detrimental and may lead to the failure of gametogenesis.

BLASTP program shows that mammalian TEX11 protein has a significant similarity to *Arabidopsis thaliana* and *Saccharomyces cerevisiae* Zip4 regarding the structure. Atzip4 mutants in *A. thaliana* cause defective meiosis, implicating an essential role in Class I Crossover formation, while synapsis could still occur without AtZIP4 (Chelysheva et al., 2007). Zip4 mutation shows negative crossover interference and interfere the distribution of the remaining crossovers, promoted by an Mms4-dependent pathway, through Zip1 (Tsubouchi et al., 2006).

Although several animal models demonstrate meiotic arrest in infertility, meiotic defects in human maturation arrest remains poorly understood (Láscarez-Lagunas et al., 2020). Tex11-knockout male mice were sterile, and an arrest in late meiosis was found in tubules of *Tex11*^–/Y^ testis. Variable synaptic failure in *Tex11*^–/Y^ pachynema was observed, suggesting that TEX11 contributes to the initiation and/or maintenance of chromosomal synapsis in male meiosis (Yang et al., 2008). Immunohistochemistry analysis of testis sections from fertile male indicates that TEX11 was mainly expressed in late-pachytene spermatocytes and in round spermatids (Yatsenko et al., 2015; Sha et al., 2018; Yu et al., 2021). However, we failed to obtain the same antibody, which was used in previous research due to the discontinuation of production. Therefore, we evaluated the testicular tissues with H&E staining and immunostaining using SYCP3, γH2AX, DMC1, and PNA to distinguish the phase of spermatogenesis affected by *TEX11* mutations. Overall, the spermatogenesis of the patients arrested at the spermatocytes stage, which is consistent with previous results that *TEX11* mutations were detected in patients with complete meiotic arrest (5 of 33 patients, 15%) (Yatsenko et al., 2015).

A meiosis-specific domain (SPO22) and some TPRs (a protein–protein interaction module composed of helical turn-helix repeats) providing interaction sites for other molecules were identified in TEX11. The C-terminal of TEX11 corresponding to a TRP-like domain (458–814 amino acids) was reported to interact with SHOC1 (a novel member of the ZMM group of proteins and an ortholog of Zip2) (Guiraldelli et al., 2018), and Nijmegen breakage syndrome 1 (NBS1) (Adelman and Petrini, 2008). TEX11 may be required to be stabilized by USP26-mediated de-ubiquitination and might participate in sex chromosomes pairing (Liu et al., 2021). Western blot analysis showed that truncated protein fragment resulting from p.K286R fs^∗^5 (P9225), p.E351X (P5048), and p.N418K fs^∗^10 (P8122) severely damaged C-terminal domain interacting with proteins such as SHOC1 and SYCP2 (Yang et al., 2008), implicating a critical role of this region. Though loaded with equal amount of total proteins, we found that the expression of TEX11 protein decreased as the protein becomes shorter, indicating that the stability of TEX11 protein requires intact domains. This may explain why the shortest truncated protein (V85L fs^∗^59) cannot be detected, and the expression of other truncated proteins were lower than the full length. The mutant p.W871C (P7583) showed substitution of a single amino acid within the TPR domain, which may alter the protein function. A screen of genomic samples from 246 azoospermic men with spermatogenic failure (no sperm in semen) found a significantly high prevalence of singleton variants in azoospermic men (7.3%), suggesting that *TEX11* is required for spermatogenesis in human. However, it is noteworthy that the functional evaluation of three analogous human *TEX11* missense mutations in transgenic mouse models identified one mutation (V748A) as a potential infertility allele and two mutations as non-causative. Tex11 KI (V749A)/KO mice displayed severe meiotic defects but no complete meiotic arrest. The differential effect of this missense mutation on the fertility of mice and humans may contribute to the species-specific requirement or context, as *Tex11* is a rapidly evolving gene with only 56% protein sequence conservation between mouse and human (Yang et al., 2015).

The c.1796 + 2T > G and c.1426-1C > T are splicing mutations. The c.1426-1C > T mutation affects the family that has never been reported supporting the inheritant models of X-linked recessive pattern.

We propose that the truncation and splicing mutations severely affect the tertiary structure of the SPO22 domain and the entire protein, disrupting its function or stability. The *TEX11* mutations may disturb the formation and function of the synaptonemal complex, causing major disruption of pachytene synapsis and anaphase spindle checkpoints, which, in turn, triggers meiotic arrest, spermatocyte apoptosis, and azoospermia.

In conclusion, three frameshift mutations, two splice site mutations, one non-sense mutation, and one missense mutation of *TEX11* were identified in seven out of 479 patients of NOA (1.5%). We found that *TEX11* mutations in infertile men are important for the diagnosis of azoospermia and meiotic arrest. The gene mutation types and phenotypes may vary among different races. What is more, it might be a prognostic marker (included in genetic screening panels) for TESE of micro-TESE to predict the sperm retrieval rate or a test for the prepregnancy screening.

## Data Availability Statement

The data presented in the study are deposited in the ClinVar repository, accession number SCV001809817-SCV001809823.

## Ethics Statement

The studies involving human participants were reviewed and approved by Institutional Review Board of Shanghai General Hospital. The patients/participants provided their written informed consent to participate in this study. Written informed consent was obtained from the individual(s) for the publication of any potentially identifiable images or data included in this article.

## Author Contributions

ZJ, CYo, and CYn designed the study and wrote the manuscript. LZ, XH, and ZiZ collected the samples. CH, EZ, and NL performed the experiments. ZL designed and arranged the project with the help of ZZu. ZL and ZZu provided the clinical and laboratory support. All authors contributed to the article and approved the submitted version.

## Conflict of Interest

The authors declare that the research was conducted in the absence of any commercial or financial relationships that could be construed as a potential conflict of interest.

## Publisher’s Note

All claims expressed in this article are solely those of the authors and do not necessarily represent those of their affiliated organizations, or those of the publisher, the editors and the reviewers. Any product that may be evaluated in this article, or claim that may be made by its manufacturer, is not guaranteed or endorsed by the publisher.

## Footnotes

1
http://www.informatics.jax.org/mgihome/homepages/

2
http://www.proteinatlas.org/
