# Supplementary material for: Novel Hemizygous Mutations of TEX11 Cause Meiotic Arrest and Non-obstructive Azoospermia in Chinese Han Population
Source: Front Genet. 2021 Sep 21;12:741355. doi: 10.3389/fgene.2021.741355 (PMC8491544; doi:10.3389/fgene.2021.741355)
Supplement: Supplementary file 1 [file Table_1.DOCX]

| **Table S1. Primer sequences used for Sanger sequencing** | | | |
| --- | --- | --- | --- |
|  |  |  |  |
| Patient No. | Change in Coding DNA (NM_001003811) | Forward primers (5’-3’) | Reverse primers (5’-3’) |
| P5648 | c.1796+2T>G | CCCAAAGGCCAGTTCTAGTT | ACTGAAGCCCAGAGACATTAAG |
| P6825 | c.1426-1C>T | TTGCTATTTGTCAGTTGCTCTTTTG | CACGGTTTCTTTTTGCATTGTTGT |
| P7583 | c.2613G>T | AGAGAGGACCTCGGTTTACG | GGGTTGAGTCCTTGTGGAGAG |
| P5048 | c.1051G>T | GCTTGCAATCAGTTCTGGGAAA | ACACTTGCGAAAATCCTACTGAC |
| P8122 | c.1254dupA | TTGAGCACGGAGTGAATGAG | CAGCGATGACATTTCCCTACA |
| P8251 | c.298delG | GCCTGTAATCCCAGCACTTT | CCCTTCCAGTTTCACACTAACT |
| P9225 | c.857delA | CTGTGGGAGTGCTAGTCAGC | CTGTGAGAGTAAATGCTGAAGCAAA |
