# Supplementary material for: Novel Hemizygous Mutations of TEX11 Cause Meiotic Arrest and Non-obstructive Azoospermia in Chinese Han Population
Source: Front Genet. 2021 Sep 21;12:741355. doi: 10.3389/fgene.2021.741355 (PMC8491544; doi:10.3389/fgene.2021.741355)
Supplement: Supplementary file 2 [file Table_2.docx]

**Table S2. Primer sequences used for plasmid construction**

| Primers | primer sequence (5’-3’) |
| --- | --- |
| TEX11 kpnI MYC F | gaggatctgaagcttggtaccATGGACAATGATGATTTTTTTTCCA |
| TEX11 EcoRI MYC R | tgctggatatctgcagaattcCTAATCTGACTTGCTCCAGTAGCCA |
| 9225-F | acaccaaatattatgataggctctcaatgctgt |
| 9225-R | cagcattgagagcctatcataatatttggtgt |
| 5048-F | tgatggatcatgaaagaTaatctgttgggtttca |
| 5048-R | tgaaacccaacagattAtctttcatgatccatca |
| 8122-F | tgaactggttacacaaacattctgtggagacaag |
| 8122-R | cttgtctccacagaatgtttgtgtaaccagttca |
| 7583-F | gatggtcaagtcctgtaataccggagtacttat |
| 7583-R | ataagtactccggtattacaggacttgaccatc |
| 8251-F | agaaaattagattacattatttgcttgcaagttgct |
| 8251-R | agcaacttgcaagcaaataatgtaatctaattttct |
