# Supplementary material for: Novel Hemizygous Mutations of TEX11 Cause Meiotic Arrest and Non-obstructive Azoospermia in Chinese Han Population
Source: Front Genet. 2021 Sep 21;12:741355. doi: 10.3389/fgene.2021.741355 (PMC8491544; doi:10.3389/fgene.2021.741355)
Supplement: Supplementary file 3 [file Data_Sheet_1.docx]

**Figure S1. Multiple protein alignment of the missense mutation in the TEX11 amino acid sequence.**

The conserved tryptophan amino acid at position 871, located within the predicted TEX11 domains with multiple tetratricopeptide repeat, was changed to cystine.
