# Supplementary figures and images for: Novel Hemizygous Mutations of TEX11 Cause Meiotic Arrest and Non-obstructive Azoospermia in Chinese Han Population
Source: Front Genet. 2021 Sep 21;12:741355. doi: 10.3389/fgene.2021.741355 (PMC8491544; doi:10.3389/fgene.2021.741355)

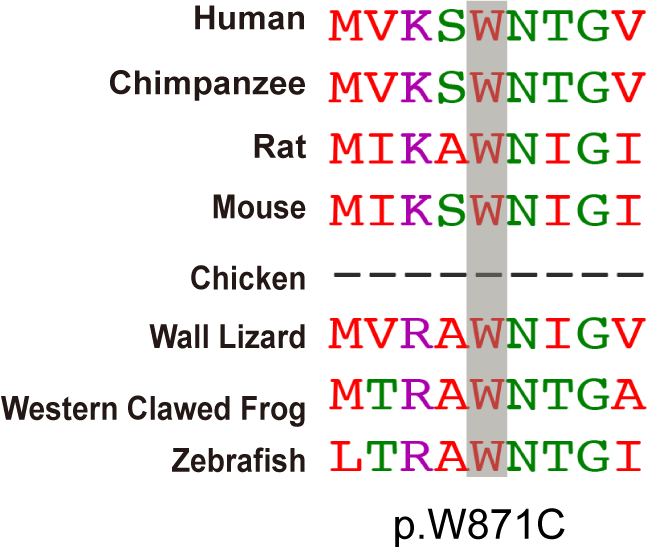

Supplement: Supplementary file 4 [file Image_1.TIF]
